# The Role of Optical Coherence Tomography Angiography in Optic Nerve Head Edema: A Narrative Review

**DOI:** 10.1155/2022/5823345

**Published:** 2022-11-30

**Authors:** Narges Karrabi, Sadid Hooshmandi, Alireza Amirabadi, Danial Roshandel, Kiana Hassanpour, Mohammad Pakravan

**Affiliations:** ^1^Ophthalmic Research Center, Research Institute for Ophthalmology and Vision Sciences, Shahid Beheshti University of Medical Sciences, Tehran, Iran; ^2^Centre for Ophthalmology and Visual Science (Incorporating Lions Eye Institute), The University of WA, Perth, WA, Australia; ^3^Department of Ophthalmology, Jones Eye Institute, University of Arkansas for Medical Sciences AR, Little Rock, USA

## Abstract

Optic nerve head (ONH) edema is a clinical manifestation of many ocular and systemic disorders. Ocular and central nervous system imaging has been used to differentiate the underlying cause of ONH edema and monitor the disease course. ONH vessel abnormalities are among the earliest signs of impaired axonal transportation. Optical coherence tomography angiography (OCTA) is a noninvasive method for imaging ONH and peripapillary vessels and has been used extensively for studying vascular changes in ONH disorders, including ONH edema. In this narrative review, we describe OCTA findings of the most common causes of ONH edema and its differential diagnoses including ONH drusen.

## 1. Introduction

Optic nerve head (ONH) edema is a funduscopic feature common to various ocular and central nervous system (CNS) disorders. Altered axonal transport at the level of lamina cribrosa is the core mechanism of ONH edema, regardless of the underlying pathology [1]. ONH edema is clinically characterized by an elevated optic disc with blurred margins. Other findings may include filling of the physiologic cup, draped retinal vessels over the margin, grayish-white peripapillary retinal nerve fiber layer (RNFL) edema with feathered margins, peripapillary hemorrhages, disc hyperemia, superficial capillary network dilation of ONH, retinal venous dilation and tortuosity, exudates, or cotton-wool spots, and retinal or choroidal folds or macular edema [2].

Although a patient's history and clinical examination may help narrow the differential diagnosis, ocular and/or CNS imaging are usually required for a definite diagnosis [3]. Visual field may reveal characteristic field defects suggestive of a specific diagnosis [4]. Magnetic resonance imaging (MRI) of the optic nerve and/or brain may show optic nerved inflammation, optic canal lesions, or space-occupying lesions [5, 6]. Posterior segment ultrasonography [7] as well as autofluorecence and enhanced depth-optical coherence tomography were also useful in detecting optic disc drusen (ODD) [8]. Optical coherence tomography (OCT) of the macula and ONH findings include spillover subretinal fluid in some patients with NAION and increased RNFL thickness in most patients with ONH edema [9].

Fluorescein angiography has been used to assess vascular changes of the ONH in different pathologies, and it is showed to be useful in detecting ONH edema in the presence of ODD [10, 11]. OCT angiography (OCTA) is a novel, noninvasive, reproducible, sensitive, contrast-free, 3-dimensional ocular imaging technology that provides structural and possibly functional information about superficial and deep retinal and choroidal vasculature [12–14]. This article aims to review the OCTA features of different causes of ONH edema and implications for its diagnosis and prognosis. The current literature involving OCTA was reviewed through Google scholar and PubMed.

### 1.1. Normal ONH and Macular OCTA

A complex vascular system originating from the central retinal artery (CRA) and posterior ciliary artery (PCA) provides the blood supply of ONH. The peripapillary capillaries are apparent most of the time, even per edema. These capillaries, derived from the CRA, are dense and spread to the macular and temporal retinal vessels. At the prelaminar and laminar regions, the papillary capillaries are also evident unless in the presence of remarkable edema. Based on the slab location, the choriocapillaris around the disc could also be visible [15].

Various artifacts may influence interpreting OCTA. Flow projection artifact from superficial blood vessels does not allow separate analysis of the flow of deep ONH [16]. Additionally, because both the disc and retinal circulations control the disc flow measurements, OCTA cannot differentiate between the PCA and retinal circulations [17].

The main features of optic disc edema in OCTA are (1) tortuosity and dilatation of the surface capillaries including ON and radial peripapillary capillaries; RPC, similar to “tangled ball or bushy”; (2) potential dropout in very severe edema or chronic stages due to compression or becoming undetectable by the slow flow; and (3) increase in sectoral and average RNFL thickness [11, 15, 18].

### 1.2. Anterior Ischemic Optic Neuropathy

Anterior ischemic optic neuropathies (AION) are classified as arteritic (AAION) and nonarteritic (NAION). AAION is frequently associated with giant cell arteritis, a systemic, visual, and life-threatening vasculitis [19]. NAION mainly threatens patients with cardiovascular risk factors and those with small, crowded cupless optic discs known as “disc-at risk” [20]. Essentially, the primary vascular pathology of NAION is thought to be the ischemia of the anterior lamina cribrosa and small vessels of the posterior ciliary vessels, resulting in a local infarct of the optic disc affecting small-caliber vessels.

In a study by Luisa Pierro et al., OCTA quantitative analysis demonstrated significant differences between representative cases of AAION and NAION, particularly in terms of vessel density (VD) values for RPC and SCP (*p* < 0.01), while DCP and CC were nonsignificant. On the contrary, vessel tortuosity (VT) values were remarkably reduced in AAION than NAION for SCP, DCP, and RPC (*p* < 0.01). VD and VT values were significantly lower both in AAION and NAION eyes compared to fellow and control eyes (*p* < 0.01). Furthermore, no significant changes were detected when comparing contralateral eyes comparing with controls. Hence, quantitative analysis in OCTA has demonstrated more vascular abnormality in AAION than NAION, which may be justified by more swelling of the optic disc characterizing AAION [20]; however, further studies are warranted to determine quantitative values as possible cutoff to differentiate between AAION from NAION [21].

Clinical applications of OCTA in NAION are reported in several studies investigating the retinal vessels [22–24], choroidal vasculature [22, 24], and optic disc perfusion [23, 25].

Some authors described a difference in vessel density or the flow index between NAION eyes and unaffected fellow eyes [22, 23, 26]. Studies comparing NAION, fellow eyes, and normal eyes are summarized in Table 1.

### 1.3. ONH OCTA

The most consistent changes in OCTA of patients with NAION are the morphological changes in the ONH vessels. OCTA has revealed tortuous capillaries inside or nearby the optic disc in NAION, which clinically is known as pseudoangiomatous hyperplasia [65]. Vessel tortuosity is a sensitive parameter for quantifying perfusion impairment occurring at the early phase of AAION and NAION and a good prognostic factor in patients with AION [20]. Moreover, irregularity, twisting, and focal loss of the superficial RPC vasculature have been reported in NAION [42].

Different studies have noted the change of the RPC visualization, vascular dropout, and fading of the regular peripapillary pattern in the acute phase of NAION (Table 1) [21, 22, 26, 35, 65]. Temporal [22] and superior [23] peripapillary sectors are mostly affected. Disc edema or hemorrhage may influence signal attenuation and may cause peripapillary vasculature dropout as a masking artifact, so it is emphasized that a decrease of the flow density at different layers in patients with NAION may not necessarily suggest a primary ischemic process but may result from compressive edema or imaging artifacts (signal attenuation by blood or imaging artifacts or edema).

Two distinct patterns of vasculature loss in NAION have been illustrated: (1) diffuse loss of microvasculature network around the optic disc and (2) additional sectoral choroidal vasculature dropout extending from the disc [66]. Lower metabolic requirements following NAION lead to the decrease of retinal blood flow as an autoregulatory response. This mechanism can be quantified through the analysis of vessel density and flow index [23, 26, 36, 67]. Vessel density is the proportion of the total measured area occupied by the vessel area. It has been well demonstrated that vessel density reduction is secondary to the loss of attributing layers of the neural tissue in the progression course of NAION [23, 36, 38, 39]. A recent meta-analysis recruiting fourteen published studies showed significantly lower vessel density in RPC, the whole enface, RPC inside the disc, and RPC peripapillary measured by OCTA in patients with NAION compared to healthy subjects. The axoplasm blockage and subsequent RNFL edema leading to impedance in the flow of RPC and retina predominate in the peripapillary region [40].

Interestingly, vessel density changes can be reversible. The superficial vessel density is reduced due to the swelling of RNFL and early thinning of the underlying RNFL layers [68, 69]. So, it should be kept in mind that part of this vascular density reduction and its reversibility is a result of the artifact caused by optic disc edema during the acute phase of disease. Vessel density might also have prognostic value in AION patients [20].

The flow index is calculated as the average flow signal in the area measured. A significant decline has been shown in the adjusted flow index at the ONH and RPC in the NAION eyes [70]. The vessel length density and the number of vessel intersections could be significantly decreased at the ONH, RPC, and vitreous layers. Peripapillary microvasculature damage is reported in both ONH and RPC layers in patients with NAION [70].

Both thinning and pachychoroid in these patients have suggested that choroidal architecture might play a role in the pathogenesis of NAION [71, 72]. While there is no difference in the vessel density at the level of the choriocapillaris, increased choriocapillaris perfusions have been noted [38, 41]. Increased signal penetration due to the atrophy of overlying RNFL and ganglion cell complex-inner plexiform layer (GCC–IPL) might explain this finding. Moreover, it may be a compensatory mechanism for the peripapillary vascular impairment in NAION.

### 1.4. Macular OCTA

Controversial results on alterations of macular vessel density have been reported. Liu et al. showed a gradual decline in the whole GCC and the corresponding whole superficial vessel density in NAION after 1-2 weeks, which deteriorated at 1-2 and 3-6 months of follow-ups along with the exacerbating superior hemifield defect [44]. Aghsaei Fard and coworkers reported a significant decline in superficial but not deep macular vessel density in NAION compared with control eyes [37]. Unlike peripapillary capillary density, progressive loss of vessel density in chronic NAION was not observed in the macular area [45]. Early decline of superficial and deep vessel density but not GCC was observed in the acute phase of NAION within two weeks of presentation [48]. Moreover, an early decrease of the whole deep vessel density has been reported in acute NAION. It is presumed that the deep capillary vortexes [73] may compensate earlier for the lower blood flow and the hypoxic/ischemic alterations of the macula, as previously explained in patients with diabetes mellitus and hypertension [74, 75].

A 6 × 6 mm scan has limited ability to discriminate vascular depth and may overestimate the parameters due to the blending of superficial and deep vessel density changes in NAION [69, 76]. Contrary to previous studies using 6 × 6 scan, evaluation with a 3 × 3 mm scan showed insignificant change in the whole deep vessel density with time. This finding can be explained by enhanced visibility of deep retinal vessels due to aggravated thinning of inner layers [44]. Nevertheless, despite the high accuracy of the 3 × 3 mm scans, its limited scanning area could miss changes outside the scanned area and underestimate the measurements [44].

OCTA has been suggested as a helpful tool for monitoring NAION progression [21, 43]. Initial data suggest that OCTA may show spontaneous, partial recovery of peripapillary vascular flow densities as the natural course of NAION in accordance with the partial improvement of the visual function. [22]. The whole vessel density of the ONH might be significantly lower in the chronic NAION compared with the acute NAION [26]. While there was no significant change in the microcirculation of the superficial peripapillary retina, the vessel density of the deep optic disc may decline significantly [26, 42]. Of note, a greater loss of superficial RPC was demonstrated suggesting a sectoral reduction of RPC as the optic disc edema subsided [44]. Although there is a significant reduction in RPC density in acute and chronic NAION compared to healthy eyes, peripapillary capillary density decreases significantly from the acute to the chronic phase of NAION [45]. Moreover, there might be no remarkable difference in optic disc blood flow area, outer vessel density, and flow index in chronic AION compared to normal eyes [77].

Contrary to these findings, Song et al. [26] and Rebolledo et al. [46] have reported no significant difference in declined peripapillary vessel density in the acute and chronic NAION. However, the evaluations were performed with a smaller scanning area [46]. It seems that longer follow-up intervals corresponding to the resolution stage can discover a significant decrease in capillary density [44]. Various degrees of axonal loss may be better demonstrated in the chronic stage when the masking artifact of edema is resolved because an artifact promoted by the edema might reduce the peripapillary vascular flow impairment [47, 78]. Also, there might be a vicious cycle of neural-vascular interaction that leads to reduced vascular supply demand in the atrophic stage of the NAION [78, 79].

### 1.5. Structure-Function Correlation

There is a good correlation between the perfusion and the visual acuity, visual field defect, and structural OCT repercussions [42]. Correlation between the decrease of the peripapillary vessel density and the location of visual field defects as well as peripapillary RNFL thinning has been reported. The whole and temporal peripapillary vessel density strongly correlated with visual acuity and temporal peripapillary superficial retinal microvasculature dropout on the ONH mode was associated with visual acuity loss [23]. This was in agreement with the previous knowledge that the extent of papillomacular bundle damage is responsible for the severity of loss of visual acuity following an episode of NAION [80]. Of note, the whole and temporal vessel density at the optic disc on the RPC mode was not correlated with visual acuity, while the whole and temporal vessel density of the peripapillary superficial retina was significantly associated with logMAR visual acuity [26].

Despite previous dye angiographic studies, OCTA revealed a hypoperfusion in the RPC and PPC following NAION, especially at the level of choroid, corresponding to both functional and structural impairments. Of note, a 100% correlation was detected between hypoperfusion at the level of the RPC and atrophy of the GCC, and a 90% correlation was detected between RPC hypoperfusion and visual field deficits. The authors hypothesized that RPC hypoperfusion is most likely to be a downstream result of the ischemic event rather than the initial component of ischemia. Hypoperfusion at the level of the peripapillary choriocapillaris was strongly correlated (80%) with GCC atrophy much like at the level of the RPC. Moreover, global peripapillary choriocapillaris hypoperfusion was more frequent than discrete, localized hypoperfusion. [24].

Irreversible vascular damage may lead to profound perfusion decrease, not affecting the overall ONH but in selective quadrants. This irreversible damage might have a role in vessel density decrease in the early stages and then RNFL loss and visual field defects in later stages [20]. Temporal peripapillary vessel density was correlated with final VA and VF outcomes in the acute NAION. In the chronic NAION, both peripapillary and superficial macular vessel densities were positively associated with visual outcomes. The reduced nasal perifoveal vessel density in superficial capillary plexus also significantly correlated with the poor visual outcomes for acute and chronic stages [81]. Temporal [22] and superior quadrants [24] have shown the most reduction in vessel density, which is compatible with the commonly identified inferonasal field defect [82]. The presence of a watershed area in temporal site of the ONH makes this part prone to ischemic injury.

## 2. Papilledema

### 2.1. ONH OCTA

In the setting of papilledema, the determination of disc swelling onset is imprecise, which can cause a bias toward OCTA features of papilledema. A “tangled ball of vessels” at the surface of the ONH has been explained [35], whilst there was no change at the level of the RPC. The visibility of the peripapillary vasculature might be enhanced in chronic papilledema as a result of increased vessel diameter and density [65].

The main differentiating feature of papilledema from NAION on ONH OCTA is the vascular dropout observed in NAION [17]. In a retrospective study investigating microvasculature changes in eyes with disc edema, peripapillary capillary network changes were noted in NAION and papillitis, whilst superficial optic disc vessel dilation and tortuosity without any peripapillary network pattern alteration were reported in patients with papilledema. [35]. The diffuse loss of microvasculature cuff without focal deficit or hypoperfusion in eyes with papilledema suggests a reduced peripapillary capillary network visibility secondary to disc edema rather than the actual ischemic process [66]. A possible role for autoregulatory vascular mechanisms has also been proposed [77].

Inaccurate autosegmentation, difficulty in recognizing the scleral canal boundaries, and inability to separate large vessels from capillaries are some of the limitations of OCTA angio analytics software when evaluating papilledema. Fell et al. developed a custom digital subtraction analysis software that successfully separated large vessels from capillaries and calculated mean perfused large vessel density and perfused capillary density in RPC, ONH, and vitreous layers. Customized OCTA postprocessing software showed a remarkable decrease in perfused capillary density in high-grade papilledema and subsequent optic atrophy. Large vessel analysis of ONH and vitreous layers may exhibit alterations in the visibility secondary to changes in ppRNFL thickness [83].

Aghsaei Fard et al. showed significantly lower peripapillary vasculature measures in papilledema and pseudopapilledema eyes than in healthy eyes and comparable measures between papilledema and pseudopapilledema. However, peripapillary “capillary” density of papilledema eyes was not significantly different from healthy subjects, whilst pseudopapilledema eyes had substantially lower capillary values than control eyes. In pseudopapilledema eyes, capillary density was considerable lower in the whole image and nasal sector peripapillary of the inner retina than in papilledema eyes. The whole image and nasal peripapillary sector capillary densities may have a diagnostic value for distinguishing actual and pseudo-disc swelling using OCTA [50].

In another study, Aghsaei Fard et al. using commercial software showed that NAION eyes had lower peripapillary total vasculature density values, followed by papilledema eyes and control eyes. The customized software showed substantially lower perfused capillary density of NAION eyes than papilledema eyes, but there were no significant differences between papilledema and control subjects. Moreover, eyes with optic neuritis had a significantly lower whole image and perfused capillary density than papilledema. However, NAION and optic neuritis were comparable using the customized software. The area under the receiver operating curves for differentiating NAION from papilledema eyes and optic neuritis from papilledema eyes gave the highest values for the whole image capillary density with the customized software, followed by peripapillary total vasculature with commercial software.

### 2.2. Macular OCTA

Macular and parafoveal vessel densities showed no significant difference between patients with papilledema and healthy controls. The whole superficial and deep macula vasculature were significantly lower in eyes with NAION compared with eyes with papilledema. Regarding GCC thickness, no significant differences were observed among NAION, papilledema, and control eyes. Whole superficial and deep macular vasculatures were correlated with visual field mean deviation but not macular GCC thickness [48]. However, the stage (acute or chronic) and/or severity of the disease play key roles in macular OCTA findings in these conditions. Further studies with a quantitative analysis of perfusion density may aid in determining the role of vascular changes in the progression of papilledema.

### 2.3. Pseudopapilledema, Optic Nerve Head Drusen, and Papillitis

There are reports that morphologic characteristics and peripapillary RNFL thickness on SD-OCT can help differentiate ONH drusen and edema [84, 85]. However, SD-OCT mainly illustrates the presence of ONH drusen and cannot rule out the concurrent disc swelling. Fluorescein leakage from the optic disc differentiates true disc edema in these cases [11].

Studies using OCTA have reported focal capillary attenuation [18] and significantly lower vessel density in ONHD compared with normal eyes [52]. Qualitative assessment of ONH microvasculature was fruitless in precise differentiation of mild disc edema (NAION) from pseudoedema (ONH drusen) due to poor intergrader agreement on grading for vessel dilation and tortuosity. Using en face images, a significant reduction in ONH VD values was noted in eyes with acute NAION compared with ONHD and healthy eyes [17]. Additionally, in the study by Cennamo et al. [52], vessel density in ONHD was significantly lower than the normal eyes. The reduction could be attributed to the lower RNFL in the adjacent area. However, this decrease was not consistently seen in the parameters of RPC layer. Therefore, the quantitative OCTA of disc microvasculature, particularly in the optic nerve head slab, may differentiate optic disc edema due to NAION from pseudo disc edema due to ONH drusen [17].

The only study investigating RPC in the acute phase of papillitis concluded that the most useful differentiating feature of papillitis from NAION was the lack of vascular dropout in papillitis. Even in the presence of significant edema that may mask the peripapillary network, they reappear beyond the edema. Quantitative studies noted vascular dilation as a consequence of inflammatory processes, which may be helpful in the diagnosis of papillitis [35].

### 2.4. Optic Neuritis

Optic neuritis (ON) is unilateral or bilateral inflammation of the optic nerve due to various causes, including multiple sclerosis (MS), optic neuritis associated with neuromyelitis optica (NMO), infectious, or isolated. In patients with MS, ON is the presenting symptom in 25% and occurs in 75% of cases during the disease course [86]. Atrophy of the RNFL occurs after episodes of ON. Since the choroid supplies blood to the retina and optic nerve, the evaluation of peripapillary choroidal thickness and vascular perfusion can be helpful in the diagnosis of optic nerve diseases [87]. Radial peripapillary capillary and choroidal slabs are two acceptable slabs to assess perfusion of the RNFL layer vessels and choroidal vessels [88].

Wang et al. used OCTA to measure the ONH flow index (defined as the average flow signal within the whole ONH) in 52 eyes of MS patients, 14 eyes with a history of ON, and 21 eyes of healthy control. They classified the flow index based on standard deviation (SD) into three categories: normal (<1.65 SDs under average), borderline (1.65 to 2.33 SDs under average), and abnormal (>−2.33 SDs lower the average). The ONH flow index was significantly reduced in patients with MS with a history of ON than healthy controls (43% vs. 5%, *p* < 0.01). 21% of patients with MS without a history of ON eyes had a reduction in flow index compared to HCs (*p*=0.198). In the parafovea zone, there was no difference between the three groups. They concluded that OCTA can be used to detect and monitor ONH perfusion in MS patients [29].

Similarly, Spain et al. investigated 68 eyes from MS patients and 55 healthy eyes with OCT and OCTA to assess the structural and vascular change in the peripapillary area. Eyes from MS patients, whether they had a history of ON or not, showed a decrease in the ONH flow index and OCT structure parameters like NFL thickness than healthy control. These parameters were decreased more in patients with ON than without ON. They compared the results with their previous study in which the ONH flow index did not have any reduction in MS without the ON group, and so due to the larger sample in this study, there was a 5.5% reduction in MS without ON compared to healthy control and 14.7% reduction in MS with ON than healthy control eyes. In conclusion, OCTA can detect optic nerve hypoperfusion and flow index decline in MS eyes. Combining these data with structural OCT parameters may increase the accuracy of the assessment of the nerve damage [89].

Summarizes studies reporting changes in OCTA parameters during ON [27, 28, 30–33, 37, 90].

### 2.5. Toxic/Nutritional, Traumatic Optic Neuropathy

Toxic optic neuropathy is an optic nerve damage due to a variety of toxins. The toxins are derived from foods, drugs, metals, and carbon dioxide. There are various signs and symptoms like bilateral visual loss, color vision reduction, and central and cecocentral scotoma due to damage to the optic nerve, retina, and chiasma. More important toxins are methanol, quinines, isoniazid, ethambutol, linezolid, tobacco, vincristine, cyclosporine, and amiodarone. Systemic conditions like diabetes and thyroid disease can make toxic substances and affect the optic nerve [91, 92]. Several modalities have been used for the diagnosis and follow-up of the patients. OCT is used to detect the thickening of the RNFL in the acute phase and thinning and atrophy in the late stage [93]. Fluorescein angiography can also evaluate retinal vascularity in toxic neuropathies but is not always accessible because of various side effects of dye-like anaphylaxis, nausea, and renal impairment.

Abri Aghdam et al. evaluated patients with NAION, traumatic optic neuropathy (TON), methanol induced optic neuropathy, compressive optic neuropathy, and healthy controls. The authors measured disc area, cup/disc ratio and cup volume, RPC vessel density inside the disc, peripapillary RPC vessel density, and peripapillary RNFL thickness. They found a significant positive correlation between RPC vessel density and RNFL thickness, and both parameters reduced in patients of all groups compared to controls. RPC vessel density can be considered as a marker of axonal energy demands and its implication for RNFL thickness reduction. Inside RPC vessel density and peripapillary RPC vessel density showed significant correlation only in NAION (*r* = 0.36, *p*=0.042) and methanol-induced optic neuropathy (*r* = 0.42, *p*=0.018). Disc area and the cup size were larger in methanol-induced optic neuritis, and RNFL thickness was lower in traumatic optic neuritis compared to other groups. Since they did not find specific vascular changes which can differentiate between groups, they concluded that structural assessment might be superior to vascular parameters in differentiating toxic and traumatic optic neuropathies [56].

Montorio et al. found no differences in vessel density of superficial capillary plexus in the macula of patients with traumatic optic neuropathy 48 hr after trauma, but a significant reduction happened in the 1st and 3rd-month, which was stable at the sixth month. The RPC area declined in the third month and was stable in the sixth month. Deep capillary plexus VD decreased 48 hr after trauma and then increased in the first month of follow-up. In the third and sixth months, the result was similar to the control group. BCVA had slightly decreased 48 hr after the trauma, but it was not significant, and follow-up examinations showed no significant difference between traumatic eyes and control. Hence, OCTA may be useful for monitoring the course of vascular changes in traumatic optic neuropathy, even in patients with stable BCVA [57]. Studies on the effect of venom envenomation [53] and iron deficiency anemia [54] on vascular changes in ONH OCTA are summarized in Table 1.

### 2.6. Leber Hereditary Optic Neuropathy

Leber hereditary optic neuropathy (LHON) is a mitochondrial disorder. Symptoms mainly occur in the second and third decade of life with vision loss and optic disc hyperemia, followed by the pallor of optic disc and atrophy in chronic phases. Studies using OCTA showed that reduction in vessel density precedes structural changes and increases from subacute to chronic phases of LHON [58, 60, 64].

## 3. Conclusion

Briefly, despite the limitations of OCTA, it is recommended as the initial test for the vascular assessment in NAION. If OCTA did not confirm the diagnosis, more invasive modalities like FA could be performed. Moreover, OCTA could be a helpful tool for quantifying and monitoring ischemia in NAION. So far, prelaminar and laminar vasculature has not been assessed due to the limitations of the technology, which may be more associated with the ischemia of PCA. It seems that the current role of OCTA in NAION is more supportive rather than diagnostic. Future research could potentially enable the diagnostic capability of quantitative measures. OCTA is helpful in differentiating NAION and papillitis from papilledema. The most significant diagnostic accuracy is related to whole-image capillary density for differentiating disc swelling. [51].

In the future, longitudinal studies with more cases will provide standard values for differential diagnostic and therapeutic goals.

OCTA can be a valuable modality to evaluate the vascular changes in ON patients to monitor and distinguish disease severity.

## Figures and Tables

**Table 1:** The role of optical coherence tomography angiography in differentiating causes of optic nerve head edema; review of current literature.

Authors	Disease	No. of cases	OCTA device	Outcome	Limitations
Lee et al. [27]	Optic neuritis	31 ON-affected eyes 31 fellow eyes 33 HC eyes	Cirrus	Reduction in parafoveal and peripapillary VD in the ON-affected eyes compared to fellow eyes	(i) Small sample size (ii) Lack of subgroup analysis (iii) A retrospective, cross-sectional study (iv) Likelihood of quantification bias

Ulusoy et al. [28]	Optic neuritis	20 patients with RRMS 24 HC	Optovue	Reduction in VD of the superficial plexus (i) Superior hemisphere (ii) Inferior hemisphere (iii) The whole image (iv) Parafoveal and perifoveal reduction in VD of the optic disc in the inferior and temporal sector	(i) Projection artifacts of OCTA images (ii) Small study group

Spain et al. [29]	Optic neuritis MS	68 eyes of 45 MS patients 55 eyes of 32 HC	Axsun technology	Reduction in ONH-FI, GCC thickness, and NFL thickness in MS patients without ON and MS patients with ON eyes	(i) Investigation of vascular risk factors using self-reports, and medication review (ii) Lack of visual field testing (iii) Lack of an automated focus in device (iv) Motion artifacts of OCTA images

Lanzillo et al. [30]	Optic neuritis MS	50 MS eyes 46 HC	Optovue	Reduction in VD in retina in MS patients with and without ON compared to healthy eye.	Small sample size

Wang et al. [29]	Optic neuritis MS	52 MS eyes 21 HC eyes	Axsun technology	Reduction in ONH-FI of the MS with ON No difference mean of parafoveal FIs among MS with ON, MS without ON, and HC eyes	Small sample size to the weak signal of some eyes

Fard et al. [31]	NAION Optic neuritis	35 demyelinating ON eyes 33 eyes with NAION 81 HC eyes	Optovue	Thinner RNFL thickness in NAION than ON eyes reduction in peripapillary VD values in NAION and ON eyes compared with HC No difference in all VD parameters between ON and NAION eyes	Small sample size clinical distinction between NAON and ON unable to determine cause-effect between VD and RNFL due to patients evaluation at the one-time point

Khader et al. [32]	Optic neuritis MS without optic neuritis	10 ON eyes 10 MS-ON eyes 10 HC eyes	Cirrus	Reduction in the average thickness and all quadrants, notably, the temporal quadrant RNFL thickness and GCL thickness in all quadrants in ON Lower superficial and deep VD index in ON and MS without ON	Motion and projection OCTA artifact loss of focus artifact and falsely reduced VD due to MS patients' ocular dysmetria OCTA cannot provide a quantitative evaluation of the flow velocity, vessel morphology, or changes in the vessel barrier

Lee et al. [27]	MS NMOSD	23 MS patients with ON (36 eyes) 37 NMOSD patients white ON (47 eyes)	Cirrus	(i) Reduction of VD of the superficial radial capillary plexus and RPC in NMOSD (ii) Association between RNFL and macular GCIPL thickness with peripapillary and parafoveal VD in the MS (iii) Association between RNFL thickness with peripapillary and parafoveal VD in NMOSD (iv) Associations between VF and VA and parafoveal and peripapillary VD in the MS group according to perfusion-visual function relationship (v) Association between the functional parameters and peripapillary VD in the NMOSD group	(i) A retrospective, cross-sectional study (ii) The likelihood of quantification bias

Rogaczewska et al. [33]	NMOSD MS	13 NMOSD patients 40 MS patients	Optovue	(i) Reduction of RPC VD in the superior and inferior sectors in NMOSD (ii) Reduction in the temporal sector of RPC in the MS group (iii) Thinner RNFL in the inferior and temporal quadrants in NMOSD (iv) No difference in the macular capillary plexuses and the GCC thickness between NMOSD and MS	Small group of NMOSD patients including only AQP4-IgG seropositive NMOSD patients. Not generizable to seronegative NMOSD

Pierro et al. [20]	AION	30 AION eyes 15 HC eyes	Topcon	Lower VD and vessel tortuosity values in AAION and NAION eyes Reduction in RNFL in AAION and NAION eyes	(i) The possible artifacts affecting OCTA images (ii) The possible effect of disc swelling on the detection of blood flow signal

Chen et al. [34]	AAION NAION ON CON TON GON	1 AAION 3 NAION 3 ON 1 CON 1 TON 1 GON	Optovue	(i) A decrease in peripapillary VD regardless of the etiology (ii) Correlation between the peripapillary vessel loss with the areas of RNFL thinning on OCT	(i) Disc edema from acute AION obscures the analysis of the peripapillary capillaries (ii) Focus on chronic optic neuropathies

Rougier et al. [35]	NAION Papillitis Papilledema	Eight eyes of 4 NAION 12 eyes of 6 papillitis 25 eyes of 13 papilledema	Cirrus	(i) NAION or papillitis: Disappearance or moderate pattern alteration in peripapillary capillary vessels (ii) Papilledema, dilation, and tortuosity of the capillaries at the surface of the optic disc, with no peripapillary network pattern changes (iii) NAION—no differences in VD and capillary flux index with unaffected fellow eyes (iv) Papillitis with higher FI in inflammatory eyes with no differences in VPD	—

Song [26]	NAION	41 eyes of 30 NAION 30 HC eyes	Optovue	(i) Reduction of VD in the peripapillary superficial retina and optic disc (ii) Lower whole VD of the optic disc in the chronic NAION than acute NAION (iii) Reduction of the VD peripapillary superficial retina and optic disc in unilateral involved eyes as compared to the fellow eyes	—

Sharma et al. [22]	NAION	6 eyes of 5 acute NAION 19 HC eyes	Optovue	Global reduction of the mean peripapillary and the peripapillary choroid layer flow density	(i) Small sample size (ii) The scarcity of NAION in Singapore (1.08/100 000)

Hata et al. [23]	NAION	15 NAION eyes 19 HC eyes	Optovue	(i) Reduction in the VD of the peripapillary retina and inside the optic disc (ii) Association between the severity of visual field defect and RNFL thinning with peripapillary VD but not with the optic disc VD (iii) Reduction in RNFL and peripapillary VD, in the superior sectors, corresponds to the VF defect.	(i) Small number of patients with NAION (ii) The inclusion of bilateral cases (iii) The lack of information on blood pressure, which could affect the intraocular perfusion pressure (iv) Evaluation in the atrophic phase of NAION (v) OCTA artifacts

Balducci et al [21]	AION	4 NA-AION patients 1 AAION patient	Optovue	Reduction of sectorial peripapillary VD and ONH and RPC VDs	OCTA images from a patient were excluded due to motion artifacts Small sample size

Liu et al. [36]	NAION	13 NAION patients 18 HC eyes	Optovue	Lower peripapillary VD The parafoveal VD was not significantly different	Ethnic groups difference Interpersonal changes in ONH blood supply

Higashiyama et al. [37]	ON	7 ON patients 7 HC eyes	Optovue	Retinal thicknesses reduction prepapillary and in the macula retinal perfusions reduction prepapillary and in the macula	Retinal perfusion showed just after treatment Detecting the slight decrease in retinal perfusion is difficult Short time between examination and treatment

Aghdam et al. [17]	ONHD NAION	10 eyes with ONHD 10 eyes in acute NAION phase 10 HC eyes	Optovue	In ONH en face images, the difference in the VD measurements between the three groups In RPC en face image, the difference in the VD measurements between three groups except nasal prepapillary section	Groups are not matched for age OCTA artifacts

Mayes et al. [24] Ten17	NAION	10 eyes of 9 NAION patients	Optovue	Flow impairment in RPC compliant with the structural defect of RNFL and GCC and VF Flow disturbance in the PCC corresponded to structural defect of the RNFL, VF, and GCC	Error and bias which result from qualitative analysis that performed by an independent reader Small sample size No longitudinal follow-up

Ling et al. [25]	NAION	21 eyes of NAION patients 19 HC eyes	Optovue	Increase nonperfused area of the optic disc A relative correlation between the VF mean defect and the nonperfused area of the optic disc	Not age-matched Excluded patients with acute-phase NAION that the optic discs exhibit constant edema Optic disc abnormal morphologies were excluded

Augstburger [38]	NAION	26 eyes of NAION patients 24 HC eyes	Optovue	Reduction in the RPC wiVD and the cpVD and correlation with RNFL thickness and visual acuity and mean deviation of the VF Capillary rarefaction in the SCP and DCP and correlation with VA	—

Wang et al. [39]	NAION	37 NAION eyes with 30 uninvolved contralateral eyes 27 HC eyes	Optovue	Lower in RNFL thicknesses Lower GCC Larger general loss volume values and sectoral loss volume Lower superficial and deep VD Correlation between RNFL thickness and VF loss	OCTA is weak to show choroidal vessels structure Measurement errors in density of choroidal vascularity

Ling [40]	NAION	14 published studies	—	Reduction in the radial peripapillary capillary whole enface vessel density RPC inside disc vessel density RPC peripapillary vessel density Peripapillary retinal NFL thickness and GCC thickness in macular zone	The consolidated estimates must be interpreted carefully as high heterogeneity is in this meta-analysis Small sample size

Dhiman et al. [41]	NAION	14 eyes of 14 NAION patients 12 HC eyes	Cirrus	Reduction in the mean total superficial retinal vessel in peripapillary area and perfusion density and the also superficial retinal perfusion	OCTA artifacts Cross-sectional study which cannot show the post-NAION vascularity changes months after the episode

Rougier et al. [42]	NAION	10 eyes of 10 NAION patients	Cirrus	A delayed ONH filling Loss of the radial aspect in peripapillary network Severe loss in peripapillary vascularization	Lack of quantified data

Fard et al. [43]	NAION POAG	31 chronic NAION 42 moderate and severe POAG 77 HC eyes	Optovue	Temporal and superior PCD were more affected Higher PCD value in inferior sector in POAG eyes Correlation between inferior and superior PCDs and also corresponding RNFL thicknesses	The PCD and RNFL are calculated with different instruments Skeletonization of capillaries did not performed during processing of image Measuring of the vessel was not accurate Cross section design of study

Liu et al. [44]	NAION	21 affected eyes 19 unaffected eyes 21 NAION patients	Optovue	Reduction in wRPC at enrollment Reduction in wRPC in NAION eyes from the acute phase to the atrophy phase with severe reduction of sRPC	VF loss of threshold was not analyzed by detail prelaminar and laminar vasculature was not evaluated

Fard et al. [45]	NAION	16 aNAION eyes 40 HC eyes	Optovue	Lower RPC densities and whole image in NAION Lower RPC densities at cNAION than aAION Lower hemi-superior and hemi-inferior RPC densities in cNAIO than aNAION Total thickness of macular GCC is less in the cNAION group No difference in total macular vessel density between groups Deep macular vessel density was not affected in NAION groups and control	OCTA artifact Initial involvement before visiting cannot be identified

Rebolleda et al. [46]	NAION	6 NAION patients 10 HC eyes	Cirrus	Reduction in the PCD, vessel density, and PD at acute and atrophic phase A significant reduction in PCD at the temporal area at the inner circle in vessel density and PD and GCIPL thinning after the 3 months	Lack of quantitative date OCTA artifacts

Contreras et al. [47]	NAION	All patients with NAION between April 1, 2004 to March 31, 2006	Stratus OCT	Lower RNFL thickness in cNAION than aAION The decrease in RNFL thickness of superior quadrant was significantly higher after 6 months No correlation between VA, VF mean density, or RNFL thickness of the affected eyes	—

Fard et al. [48]	NAION Papilledema	20 eyes with NAION 39 eyes with papilledema 22 HC eyes	Optovue	Vascular density is lower in the macular and parafoveal area in superficial and deep slabs in NAION eyes VD values are similar in papilledema and control eyes Superficial and deep macula vascular density is lower in the NAION than papilledema GCC thickness was similar in all groups	The cross-sectional design of study OCTA artifact

Bilen and Atilla, [49]	IIH	38 eyes of 19 IIH 42 eyes of 21 HC	Optovue	Increase in inf. RNFL thickness Decrease in mean peripapillary VD	IIH patients were a heterogeneous group in diagnostic time and treatment Right and left eyes were evaluated separately, to diminish potential environmental and genetic factors which could have reduced the study's statistical power

Fard et al. [50]	Papilledema Pseudopapilledema	41 eyes of 21 papilledema 27 eyes of 15 pseudopapilledema 44 eyes of 44 HC	Optovue	Greater RNFL thicknesses papilledema eyes No differences in GCC thickness among all groups Lower peripapillary vasculature values in papilledema and pseudopapilledema Differences in the peripapillary capillary density of papilledema eyes from healthy but lower in pseudopapilledema eyes than nasal sector peripapillary capillary density of inner retina and other whole image in pseudopapilledema	A difference in segmentation of irVD and trVD images for the control group may impact VD for the whole image analyses of custom software versus papilledema and pseudopapilledema in the ONH secondary to disc edema

Fard et al. [51]	NAION Papilledema Optic neuritis	29 eyes with NAION 44 eyes with papilledema 8 eyes with acute optic neuritis 48 HC eyes	Optovue	In RPC and ONH images, reduction of the VD in each sextant, and the whole peripapillary VD values in papilledema and NAION eyes, with the latter being more severely affected. No differences in VD between papilledema eyes and optic neuritis eyes Lower nasal and whole peripapillary sextant values in NAION than optic neuritis eyes for the ONH image	There was no prospective power calculation; however, when statistically insignificant results were obtained, the power was calculated retrospectively

Cennamo et al. [52]	ONHD	19 eyes of 13 ONHD 24 eyes of 16 HC	Optovue	Lower GCC, RNFL, flow index, and VD parameters in ONHD patients No visual field parameters difference between the two groups	OCTA images may have been affected by movement and blinking

Güler et al. [53]	Victims of viper bite	31 victims of viper bite 31 HC eyes	Optovue	Increase in vascular densities and foveal flow areas Decrease in flow areas and parafoveal vascular densities Increase in retinal thickness in the parafoveal and foveal regions	Do not return patients for follow-up visits Ethically inappropriate to spend OCTA shots time on these patients that require treatment and close follow-up

Korkmaz et al. [54]	Iron deficiency anemia	32 patients with IDA 30 HC eyes	Optovue	Reduction in capillary plexus density Reduction in density of SCP in the perifoveal area	Limitation of macular anatomy information in children Not reported normal values of OCTA in children

Pellegrini et al. [55]	Vitamin B12 deficiency optic neuropathy	1 patient	Topcon	Reduction in peripapillary and macular VD that correlated well with the areas of RNFL thinning	—

Abri Aghdam et al. [56]	NAION CON MION TON	32 patients with NAION 18 patients with CON 32 patients with MION 23 patients with TON 55 HC eyes	Optovue	Lower peripapillary RNFL thickness and RPC VD in all patients Correlation between the RPC VD and peripapillary RNFL thickness in normal subjects and all study groups Correlation between inside and outside disc RPC VD in the NAION and MION No difference in terms of peripapillary and inside disc VD among the groups	Extracting results from the Optovue RTVue XR may cause bias, focusing mainly on patterns, correlations, and comparisons but not raw data. To homogenize the study, patients with established optic atrophy were included in the study, but this was not ethical for CON patients

Montorio et al. [57]	Ocular blunt trauma	18 eyes of 18 patients 18 HC eyes	Optovue	Reduction in GCC and RNFL thicknesses and VD of the RPC and SCP from 1 month to 3 months after the trauma The initial decrease in the DCP VD after one month then increases until the sixth month to reach values same as those of the control group	Small sample size Absence of a longer follow-up

Asanad, et al. [58]	LHON	Case report	Cirrus	Perfusion defects in the nasal and temporal peripapillary nerve fiber layer	—

Kousal et al. [59]	LHON	12 eyes of 6 patients 6 HC eyes	Heidelberg	Decrease in the radial peripapillary microvascular network in all five affected eyes of three individuals	Due to the lack of available software, a quantitative assessment of capillary densities could not be performed. Cannot explain artifacts and variability between different examinations because of the impossibility of performing the OCTA repeatedly

Balducci et al. [60]	LHON	22 LHON patients	Optovue	Reduction of VD in the infratemporal and temporal sectors of LHON-e compared with controls and in the temporal sector compared with LHON-u Reduction of VD in the supra and infratemporal and temporal sector of LHON-I compared with controls and LHON-u Reduction of VD in all sector of LHON-ch compared with all groups	Many patients had both eyes studied, which can be a statistical confounder. VD measurement was not done in the macular region Genetic heterogeneity of the patients

Gaier et al. [61]	LHON	Case report		More prominent telangiectatic vessels and demonstrated dilated peripapillary microvasculature temporally in the inner layers of the retina Dense temporal capillaries within the papillomacular bundle	Case report

Matsuzaki et al. [62]	LHON	Case report		Telangiectatic vessels and RPC defects in the temporal section Relationship between RPC defective areas and RFT thinning with time	Case report

Takayama et al. [63]	LHON	Case report		Parapapillary telangiectatic blood vessels	Case report

Yu et al. [64]	LHON	28 eyes of 14 LHON patients 28 eyes of 14 HC	Optovue	In subacute and chronic LHON Reduction in SCP and IRT Reduction in RPC VD and thickness in the inferior temporal and temporal sectors Lower RPC VD and thickness in the inferior temporal, superior temporal, temporal and nasal sectors than the subacute stage	There was genetic heterogeneity in the patients studied Due to the rarity of LHON, the sample size was inevitably small

ON: optic neuritis; RPC: radial peripapillary capillaries; VD: vessel density; RRMS: relapsing remitting multiple sclerosis; HC: healthy control; ONH-FI: optic nerve head-flow index; GCC: ganglion cell complex:; NMOSD: neuromyelitis optica spectrum disease; NAION: nonarteritic anterir ischemic optic neuropathy; AAION: arteritic anterior ischemic optic neuropathy; GCIPL: ganglion cell-inner plexiform complex; CON: compressive optic neuropathy; TON: traumatic optic neuropathy; GON: glaucomatous optic neuropathy; ONHD: optic nerve head drusen; MION: methanol-induced optic neuropathy; LHON: leber hereditary optic neuropathy; IIH: diopathic intracranial hypertension; PCD: peripapillary capillary density; ONHD: optic nerve head drusen; GCC: ganglion cell complex.
